# A model-based statistic for detecting molecular markers associated with complex survival patterns in early-stage cancer

**DOI:** 10.1186/2043-9113-2-14

**Published:** 2012-08-06

**Authors:** Philippe Broët, Thierry Moreau

**Affiliations:** 1 Assistance Publique-Hôpitaux de Paris, Hôpital Paul Brousse; 2 Faculty of Medicine, University Paris-Sud, Paris, France; 3, INSERM, UMR-669, Villejuif, France; 4 INSERM, U 1018, Biostatistics Team, 94807 Villejuif, France; 5, Université Paris-Sud, UMR-S 1018, 94807 Villejuif, France

**Keywords:** Clinical genomic, Survival analysis, Early-stage cancer, Cure rate model, Long-term survivors, Score test

## Abstract

**Background:**

In early-stage of cancer, primary treatment can be considered as effective at eliminating the tumor for a non-negligible proportion of patients whereas for the others it leads to a lower tumor burden and thereby potentially prolonged survival. In this mixed population of patients, it is of great interest to detect complex differences in survival distributions associated with molecular markers that potentially activate latent downstream pathways implicated in tumor progression.

**Method:**

We propose a novel model-based score test designed for identifying molecular markers with complex effects on survival in early-stage cancer. From a biological point of view, the proposed score test allows to detect complex changes in the survival distributions linked to either the tumor burden or its dynamic growth.

**Results:**

Simulation results show that the proposed statistic is powerful at identifying departure from the null hypothesis of no survival difference. The practical use of the proposed statistic is exemplified by analyzing the prognostic impact of Kras mutation in early-stage of lung adenocarcinomas. This analysis leads to the conclusion that Kras mutation has a significant negative prognostic impact on survival. Moreover, it emphasizes that the complex role of Kras mutation on survival would have been overlooked by considering results from the classical logrank test.

**Conclusion:**

With the growing number of biological markers to be tested in early-stage cancer, the proposed score test statistic is a powerful tool for detecting molecular markers associated with complex survival patterns.

## Background

Entering the era of so-called personalized oncology through the growing use of molecular markers, one of the main questions concerns their capacities to refine patient prognosis beyond classical bio-clinical risk factors. From clinically and pathologically well-defined group of patients, these markers need to demonstrate their abilities to reveal heterogeneity in survival times among patients. For patients with early-stage of cancer treated with curative therapy, the problem is particularly challenging since molecular markers often reflect complex interplay of dowstream pathways that drive either the remaining tumor burden or its dynamic growth.

Cure rate models, especially those with biological interpretation, are well-suited for analyzing such data. These models are formulated by assuming that the population under study is composed of two subpopulations of patients, those who have no persitant tumor (sometimes referred as long-term survivors or cured patients) and those who have persistent tumor burden and are susceptible of experiencing a disease recurrence. In the literature, the oldest approach relies on two-component mixture models which incorporate a cure fraction in a parametric or semi-parametric framework (for a review, see [1]). A different approach, which defines the cumulative hazard as a bounded increasing positive function and relies on a mechanistic model of cancer, has been introduced by Yakovlev et al. [2-4]. This cure rate model (sometimes referred as promotion time cure model [5]) defines the improper survival distribution whereby each individual is exposed to recurrences that arise from unobservable tumor clonogens surviving the primary treatment. A clonogen is defined as a cell (or a group of genotypically identical cells) that has the capacity to divide, disseminate and proliferate indefinitively for giving rise to local or distant tumor recurrence. Each surviving clonogen has its own dynamic growth and the tumor is detected as soon as any one of the clonogens is able to produce a clinically overt tumor. The elapsed time between the end of the primary treatment and the clinical disease corresponds to the time-to-event. Assuming relevant probability distributions for the number of (unobserved) clonogens and for the clonogenic’s time-to-event, one can deduce the marginal (or population) survival distribution. From biological considerations, the Poisson distribution has been the classical choice for the distribution of the number of clonogens [4,5]. Relying on this latter modelling assumption, marginal semi-parametric cure models have been proposed from which different statistics have been deduced to test for identity of the survival curves [6-8]. However, a limitation of the Poisson distribution, on which these models are built, is that it is not flexible enough for allowing, among uncured patients, different probability distribution of the number of surviving clonogens. In particular, if the probability of being cured (no clonogen) after the primary treatment is identical across all patients, it necessarily implies a same distribution for the number of surviving clonogens among uncured patients. In this context and from a Bayesian perspective, Yin et al. [9] have proposed a family of transformation cure models that gives more flexibility for modelling survival curves and includes the two-component mixture model and the Poisson cure model as special cases [9,10]. However, this family does not provide an easy biological interpretation regarding changes in the cure fraction, the distribution of surviving clonogens and the tumor progression.

In this work and based on an alternative mechanistic cure rate model, we propose a novel score test statistic for detecting molecular markers associated with complex survival patterns in early-stage cancer. After introducing an alternative semi-parametric cure rate model that allows to describe changes in the survival distributions linked to either the tumor burden (cure rate fraction and surviving clonogens distribution) or its dynamic growth (time-to event distribution), a model-based score test is proposed. This novel score test is designed for detecting molecular markers associated with complex survival patterns in early-stage cancer. We illustrate the clinical interest of this statistic by investigating the impact on survival distributions of genetic (Kras mutation), genomic (chromosomal aberration) and histopathologic markers among patients with early-stage lung adenocarcinoma.

## Methods

### Modeling background

Here, we focus on a binary variable which allocates the patients in two groups *i*=0,1 (with *n*_*i*_ subjects in group *i*(*n*=*n*_0_ + *n*_1_)). For each patient *j*, *G*_*j*_denotes the indicator variable of group 1. For the lung cancer dataset, this variable indicates the presence/absence of Kras mutation. In the following, a tumor is modeled as a set of clonogens, with identical properties and independent evolution. For each patient *j* in group *i*, let the random variables $T_{\mathrm{ij}}^{k}$ associated to the *k*^*th*^ latent (unobservable) clonogen, be the time-to-progression until a detectable recurrence with (clonogenic) survival function *A*_*i*_(*t*). Let *K*_*ij*_ be the number of latent clonogens that survived the treatment for patient *j* in group *i*. We suppose that for the two groups, *K*_*ij*_ is distributed with probability mass function *Φ*_0_*Φ*_1_and *K*_*ij*_ is supposed to be independent of $T_{\mathrm{ij}}^{k}$. Let denote $T_{\mathrm{ij}}^{*}=\mathrm{mi}n_{1\leq k\leq K_{\mathrm{ij}}}(T_{\mathrm{ij}}^{k})$ the time-to-event of the earliest clonogen and *C*_*ij*_ the censoring time. We assume that $T_{\mathrm{ij}}^{*}$ and *C*_*ij*_satisfy the condition of independent censoring [10]. For each subject, the data consist of $X_{\mathrm{ij}}=\min(T_{\mathrm{ij}}^{*},C_{\mathrm{ij}})$ the observed time of follow-up, $\delta_{\mathrm{ij}}=1_{\left(X_{\mathrm{ij}}=T_{\mathrm{ij}}^{*}\right)}$ the indicator of the occurence of the earliest clonogen and *G*_*j*_ the indicator variable of group 1. We also denote *Y*_*ij*_(*t*)=1_(*t*≤_*Xij*_)_the indicator of being at risk for an event at time *t*.

For each patient *j* in group *i* with *K*_*ij*_latent clonogens, the conditional (patient-specific) survival function is expressed as:

$$\begin{array}{lll} S_{\mathrm{ij}}(t|K_{\mathrm{ij}}) & =\text{Pr}\left(T_{\mathrm{ij}}^{*}>t\right)\; & \;\\ & =\text{Pr}\left(T_{\mathrm{ij}}^{1}>t,\ldots ,T_{\mathrm{ij}}^{K_{\mathrm{ij}}}>t\right)=A_{i}{\left(t\right)}^{K_{\mathrm{ij}}}\; & \; \end{array}$$

Thus, the marginal (population) survival function (for group *i*) is given by:

$$S_{i}(t)=\overset{\infty }{\underset{k=0}{\sum }}S_{\mathrm{ij}}(t|k)\underset{\Phi_{i}}{\text{Pr}}(k)=\overset{\infty }{\underset{k=0}{\sum }}A_{i}{\left(t\right)}^{k}\underset{\Phi_{i}}{\text{Pr}}(k)$$

Assuming that the number of clonogens in treated tumors is following for the two groups a Poisson distribution [2-4], the marginal distribution is such as : *S*_*i*_(*t*)=exp {−*ξ*_*i*_[1−*A*_*i*_(*t*)] } where *ξ*_*i*_ (i.e. the Poisson parameter) is the mean number of clonogens and *exp*(−*ξ*_*i*_) is the probability of having no surviving clonogen (cure fraction). From this framework, one can modelize short and long-term effects of a marker [6-8]. The short-term effect (linked to *A*_*i*_(*t*)) formulates the shape of the difference between the (clonogenic) latent survival functions. The long-term effect (linked to *ξ*_*i*_) quantifies the difference in the long-term survivors rates. It is straighforward to see that a same cure fraction between the different groups (no long-term effect) implies a same distribution for the number of surviving clonogens.

In the following, we consider a family of discrete distributions proposed by Katz [11] for which the Poisson distribution is considered as the benchmark model (null model). This family allows to consider different conditional probability mass functions for the number of surviving clonogens (${\text{Pr}}_{\Phi_{i}}(K_{\mathrm{ij}}=u|K_{\mathrm{ij}}>0)$) with a same cure fraction ${\text{Pr}}_{\Phi_{i}}(K_{\mathrm{ij}}=0)$.

### Distribution of the number of clonogens

We recall that Katz [11,12] proposed a family of discrete distributions with the property that successive count probabilities satisfy the following first-order recurrence formula:

$$\frac{\text{Pr}(x+1)}{\text{Pr}(x)}=\frac{\omega +\mathrm{\theta x}}{1+x};\;x=0,\ldots ,\infty_{+}$$

where *ω*>0 and *θ*<1.

Katz showed that the probability generating function is such as:

$$\begin{array}{c} g(s;\omega ,\theta )={\left[{\left(1-\theta \right)}^{-1}\times \left(1-\mathrm{\theta s}\right)\right]}^{-\frac{\omega }{\theta }}\;\text{for}\;\theta \neq 0\\ \;\;\;\;g(s;\omega ,\theta )=\text{exp}\;\;\left[-\omega \left(1-s\right)\right]\;\text{for}\;\theta =0 \end{array}$$

with |*s*|≤1.

It follows that the initial probability is equal to: $\text{Pr}(0)=p_{0}={\left(1-\theta \right)}^{\frac{\omega }{\theta }}$ for *θ*≠0 (*p*_0_=*e*^−*ω*^ for *θ*=0). Thus, this family allows us to consider different conditional probability mass functions (Pr(*x*|*x*>0)) with a same *p*_0_.

Moreover, it is worth noting that *ω*=*μ*^2^/*σ*^2^and *θ* is linked to the dispersion index (variance-to-mean ratio) such as : *σ*^2^/*μ*= (1−*θ*)^−1^. This family covers various distributions with the property of being under-dispersed (*θ*<0), over-dispersed (*θ*>0) or equi-dispersed (*θ*=0). This latter case corresponds to the Poisson distribution. For *θ*<0, it includes Binomial distributions (*N*=−*ω*/*θ*;*p*=*θ*/ (*θ*−1)) whereas for *θ*>0 it includes Negative Binomial distributions (*u*=*ω*/*θ*;*P*=*θ*/ (1−*θ*)).

Relying on this family of distributions, we propose to consider the following semi-parametric cure model.

### Improper survival function

According to the above results, a semi-parametric improper cure model, which encompasses the Poisson cure model, is obtained as follows:

The marginal survival function is defined such as:

$$S_{i}(t)=\overset{\infty }{\underset{k=0}{\sum }}S_{\mathrm{ij}}(t|k)\underset{\Phi_{i}}{\text{Pr}}(k)=\overset{\infty }{\underset{k=0}{\sum }}A_{i}{\left(t\right)}^{k}\underset{\Phi_{i}}{\text{Pr}}(k)$$

where ${\text{Pr}}_{\Phi_{i}}(k)$ is the Katz probability mass function and *A*_*i*_(*t*) is a decreasing function such as 1≥*A*_*i*_(*t*)≥0.

Thus, we have the following general survival functions in group *i*=0,1:

$$\begin{array}{c} \;\;\;\;S_{0}(t)=\exp\left\{-\omega_{0}\left[1-A_{0}(t)\right]\right\}\\ S_{1}(t)={\left[{\left(1-\theta \right)}^{-1}\times \left(1-\theta A_{1}(t)\right)\right]}^{-\frac{\omega_{1}}{\theta }} \end{array}$$

The corresponding cumulative hazard function and hazard function are noted *Θ*_*i*_(*t*)=−log [*S*_*i*_(*t*)] and $\lambda_{i}(t)=\frac{\partial }{\mathrm{\partial t}}\Theta_{i}(t)$, respectively. It is straighforward to see that *S*_0_(*t*) and *S*_1_(*t*) are improper survival functions with cure fractions $S_{0}(\infty_{+})=e^{-\omega_{0}}$ and $S_{1}(\infty_{+})={\left(1-\theta \right)}^{\frac{\omega_{1}}{\theta }}$, respectively. Here, *A*_0_(*t*) and *A*_1_(*t*) are arbitrary latent survival functions decreasing with time from one to zero. We can give different shapes by modeling the function such as *A*_1_(*t*)=*A*_0_(*t*,*α*) where $D_{0}(t,\alpha )=-\frac{\partial }{\mathrm{\partial t}}A_{0}(t,\alpha )$ refers to the corresponding density function and *α* is a real parameter with *A*_0_(*t*,0)=*A*_0_(*t*). In the following section, we will consider a classical log-linear relationship such as $A_{0}(t,\alpha )=A_{0}{\left(t\right)}^{e^{\alpha }}$. Thus, the parameter *α*formulates the shape of the difference between the clonogenic survival functions for group 0 and 1. When *α*≥0 (resp. *α*≤0) patients belonging to groupe 1 have earlier (resp. later) relapses as compared to group 0. Here, the Poisson model is considered as the reference one which leads to the marginal survival *S*_0_(*t*). Changes in the distribution of the number of clonogens are interpreted with regard to this model. It is worth noting that the Poisson cure model can also be considered as representing an homogeneous multi-clonogenic model and departure from this model can be interpreted as either an under-dispersed (single clonogenic model) or over-dispersed (heterogeneous multi-clonogenic model) situation.

It is useful for the following to write the ratio of the hazard functions *λ*_0_(*t*) and *λ*_1_(*t*) deduced from model (1) so that:

$$\begin{array}{lll} \lambda_{1}(t)= & \;\lambda_{0}(t)\exp\left\{\log\left[\omega_{1}/\omega_{0}\right]+\log\left[D_{0}(t,\alpha )/D_{0}(t)\right]\right)\; & \;\\ & \left(-\log\left[1-\theta A_{0}(t,\alpha )\right]\right\}.\; & \; \end{array}$$

In the following, we denote *γ*=log [*ω*_1_/*ω*_0_]. From a biological perspective, belonging to group 1 is associated with changes in the cure fraction, the conditional distribution of the number of surviving clonogens or the latent survival (tumor progression) through the parameters of interest *γ*, *θ* and *α*. If *α*=0, the latent (clonogenic) survival curves are identical between the two groups whatever the distribution of the number of clonogens. If *θ*=0, there is a same probability distribution family (Poisson) for the number of clonogens whatever the dynamic of the clonogens (*α*) or the cure fraction (*γ*). This latter case corresponds to the classical Poisson cure rate model. If *θ*=*α*=0, it corresponds to the proportional hazards hypothesis whereby the relative risk is constant over time but the improper survival distributions converges to different cure fractions. Moreover, it should be noted that using a different parametrization and constraining the quantity *θ*/*ω*_1_to lie on [0,1] leads to the transformation cure model [9].

In this work, the general null hypothesis to be tested *H*_0_:*θ*=*α*=*γ*=0 is the lack of survival difference between the two groups.

#### The proposed statistic

In the following, we derive a score statistic which is optimal under a classical log-linear relationship such as $A_{0}(t,\alpha )=A_{0}{\left(t\right)}^{e^{\alpha }}$ so that the ratio of the hazard functions between the two groups is such as:

$$\begin{array}{lll} \lambda_{1}(t)= & \;\lambda_{0}(t)\exp\left\{\right)\gamma +\alpha +\log\left[A_{0}(t)\right]\left(e^{\alpha }-1\right)\; & \;\\ & \left(\;\;\;\;\;-\log\left[1-\theta A_{0}{\left(t\right)}^{e^{\alpha }}\right]\right\}\;\; & \; \end{array}$$

Thus, the log-partial likelihood derived under this multiplicative model is such as:

$$\begin{array}{lll} \log L\left(\theta ,\alpha ,\gamma ;G\right)= & \;\Sigma_{j=1}^{n}\delta_{j}\left\{\;\upsilon (t_{j})G_{j}\right)\; & \;\\ & \left(\;\;-\log\left[\overset{n}{\underset{k=1}{\sum }}Y_{k}\left(t_{j}\right)e^{\upsilon (t_{j})G_{k}}\right]\right\}\; & \; \end{array}$$

where $\upsilon (t)=\gamma +\alpha +\log\left[A_{0}(t)\right]\left(e^{\alpha }-1\right)-\log\left[1-\theta A_{0}{\left(t\right)}^{e^{\alpha }}\right]$

The score vector is derived from the first derivative of the log-partial likelihood with respect to *θ*, *α* and *γ* evaluated under *H*_0_:*θ*=*α*=*γ*=0.

The score vector is deduced under the null hypothesis (*H*_0_:*θ*=*α*=*γ*=0). The three components are as follows:

$$\begin{array}{lll} {\hat{V}}_{H_{0},\alpha }\; & =\;\overset{n}{\underset{j=1}{\sum }}\delta_{j}\left[1+\log\left(1-\frac{\Theta_{0}(t_{j})}{\omega_{0}}\right)\right]\left\{G_{j}-\frac{\overset{n}{\underset{k=1}{\sum }}Y_{k}\left(t_{j}\right)G_{k}}{\overset{n}{\underset{k=1}{\sum }}Y_{k}\left(t_{j}\right)}\right\}\; & \;\\ {\hat{V}}_{H_{0},\theta }\; & =\;\overset{n}{\underset{j=1}{\sum }}\delta_{j}\left[1-\frac{\Theta_{0}(t_{j})}{\omega_{0}}\right]\left\{G_{j}-\frac{\overset{n}{\underset{k=1}{\sum }}Y_{k}\left(t_{j}\right)G_{k}}{\overset{n}{\underset{k=1}{\sum }}Y_{k}\left(t_{j}\right)}\right\}\; & \;\\ {\hat{V}}_{H_{0},\gamma }\; & =\;\overset{n}{\underset{j=1}{\sum }}\delta_{j}\left\{G_{j}-\frac{\overset{n}{\underset{k=1}{\sum }}Y_{k}\left(t_{j}\right)G_{k}}{\overset{n}{\underset{k=1}{\sum }}Y_{k}\left(t_{j}\right)}\right\}\; & \; \end{array}$$

For computing the score statistic, we should substitute *Θ*_0_(*t*) and *ω*_0_ by efficient estimators ${\hat{\Theta }}_{0}(t)$ and ${\hat{\omega }}_{0}$ computed under the null hypothesis *H*_0_. Here, ${\hat{\Theta }}_{0}(t)=\overset{n}{\underset{j=1}{\sum }}\overset{t}{\underset{0}{\int }}{\left\{\overset{n}{\underset{k=1}{\sum }}Y_{k}(s)\right\}}^{-1}dN_{j}(s)$, where *N*_*j*_(*t*)=1_{_*Xj*_≤*t*,*δ*_*j*_=1}_ is the left-continuous version of the Nelson-Aalen estimator for the cumulative hazard [13] obtained by using the pooled sample and ${\hat{\omega }}_{0}={\hat{\Theta }}_{0}(t_{\max})$ is the maximum value of this estimator computed at the last observed failure time *t*_max_. In our problem, the limiting distribution of the proposed statistic where *ω*_0_ is replaced by ${\hat{\omega }}_{0}$ is obtained by using the results of Pierce [14] in the context of improper survival distribution [8]. Here, ${\hat{\omega }}_{0}$ is an efficient estimator of *ω*_0_if the upper bound of the domain for the survival distribution is less or equal to the upper bound of the domain for the censoring distribution [8,14]. In practice, this latter condition expresses the fact that the uncured patients should experience the event within the maximum length of follow-up. This condition is assumed to be verified and is required for establishing the limiting distribution of the proposed statistic.

The corresponding information matrix $\overset{ˆ}{I}$ is such as:

$$\begin{array}{lll} \frac{\partial^{2}\log L}{\partial^{2}\alpha }\; & =\;\overset{n}{\underset{j=1}{\sum }}\delta_{j}{\left[1+\log\left(1-\frac{\Theta_{0}(t_{j})}{\omega_{0}}\right)\right]}^{2}\left\{\Delta_{j}\right\}\; & \;\\ \frac{\partial^{2}\log L}{\partial^{2}\theta }\; & =\;\overset{n}{\underset{j=1}{\sum }}\delta_{j}{\left[1-\frac{\Theta_{0}(t_{j})}{\omega_{0}}\right]}^{2}\left\{\Delta_{j}\right\};\frac{\partial^{2}\log L}{\partial^{2}\gamma }=\overset{n}{\underset{j=1}{\sum }}\delta_{j}\left\{\Delta_{j}\right\}\; & \; \end{array}$$

and

$$\begin{array}{lll} \frac{\partial^{2}\log L}{\partial \alpha \partial \theta }\; & =\;\overset{n}{\underset{j=1}{\sum }}\delta_{j}\left[1+\log\left(1-\frac{\Theta_{0}(t_{j})}{\omega_{0}}\right)\right]\left[1-\frac{\Theta_{0}(t_{j})}{\omega }\right]\left\{\Delta_{j}\right\}\; & \;\\ \frac{\partial^{2}\log L}{\partial \gamma \partial \theta }\; & =\;\overset{n}{\underset{j=1}{\sum }}\delta_{j}\left[1-\frac{\Theta_{0}(t_{j})}{\omega_{0}}\right]\left\{\Delta_{j}\right\}\; & \;\\ \frac{\partial^{2}\log L}{\partial \alpha \partial \gamma }\; & =\;\overset{n}{\underset{j=1}{\sum }}\delta_{j}\left[1+\log\left(1-\frac{\Theta_{0}(t_{j})}{\omega_{0}}\right)\right]\left\{\Delta_{j}\right\}\; & \; \end{array}$$

with $\Delta_{j}={\left[\frac{S^{\left(1\right)}(0,0,0,t)}{S^{\left(0\right)}(0,0,0,t_{j})}\right]}^{2}-\left[\frac{S^{\left(2\right)}(0,0,0,t_{j})}{S^{\left(0\right)}(0,0,0,t_{j})}\right]$

*where*$S^{\left(r\right)}(0,0,0,t)=n^{-1}\overset{n}{\underset{k=1}{\sum }}Y_{k}\left(t_{j}\right)G_{j}^{r}$ with *r*=0,1,2. 

The elements of the score vector and of the information matrix ($I_{H_{0}}$) are computed by using efficient estimators of *Θ*_0_(*t*_*j*_) and *ω*_0_as given above.

Finally, the statistic

$$S_{H_{0}}=\left({\hat{V}}_{H_{0},\alpha },{\hat{V}}_{H_{0},\theta },{\hat{V}}_{H_{0},\gamma }\right){Î}_{H_{0}}^{-1}{\left({\hat{V}}_{H_{0},\alpha },{\hat{V}}_{H_{0},\theta },{\hat{V}}_{H_{0},\gamma }\right)}^{\prime \prime }$$

is asymptotically distributed under *H*_0_as a chi-square with three degrees of freedom.

## Results

### Simulation study

We conducted a simulation study to evaluate the finite-sample performance of the proposed statistic. We reported the size of the test as well as the power properties of the proposed test (noted $S^{H_{0}}$) together with those obtained with the classical Logrank test (noted *LR*) [10].

We considered a single binary variable taking a value of 0 (e.g. absence of a marker) or 1 (e.g. presence of a marker) with half of the individuals having value 1. We assumed that the survival distribution (for group 0) is such as: $S_{0}(t)=\overset{\left[-\omega_{0}\left(1-e^{-t}\right)\right]}{\exp}$. For group 1, we investigated over/under-dispersed scenarios where *S*_1_(*t*) can be viewed as a marginal improper survival function with either Negative binomial (overdispersion) or Bernoulli (underdispersion) distributions for the number of clonogens. For overdispersion (*θ*>0), we considered cases such as : $S_{1}(t)={\left(\frac{1-\theta e^{-e^{\alpha }t}}{1-\theta }\right)}^{-\frac{\omega_{1}e^{\gamma }}{\theta }}$ with the same cure fraction ($S_{0}(\infty_{+})=S_{1}(\infty_{+})$) or different cure fractions (*S*_0_(*∞*_ + _)≠*S*_1_(*∞*_ + _)) and with/without the same latent survival function (*A*_0_(*t*,*α*)=*A*_0_(*t*)=*e*^−*t*^or *A*_0_(*t*,*α*)≠*A*_0_(*t*)). For underdispersion (*θ*<0), we considered cases such as : $S_{1}(t)=\left(\frac{1-\theta e^{-e^{-\alpha }t}}{1-\theta }\right)$ with the same cure fraction or different cure fractions and with/without the same latent survival function.

Various values for the parameters were considered. For overdispersed cases, we took *θ*=0.78 and for the under-dispersed cases we took *θ*=−1. For the baseline cure rate fraction, we took: $S_{0}(\infty_{+})=e^{-\omega_{0}}=0.30,0.50,0.70$. The values for *ω*_1_ are chosen so that the cure fractions are equal or different with *e*^*γ*^being equal to: 1 and 1.2. For the latent survival distribution shift, we considered values *e*^*α*^=1,1.25,1.5. The censoring time *C*_*j*_ was generated from an exponential distribution with parameter *ζ*. Values for *ζ*were computed from the chosen percentage of censoring and from the parameters of the considered distributions. The percentage of censoring below refers only to the percentage of censored observations without the cure fraction. We investigated no censoring and 30% censoring. The number of subjects within a group was chosen to be 100. For each configuration, 500 replications were performed and the levels and powers of the two tests were estimated at the nominal level 0.05.

**Figure 1:**
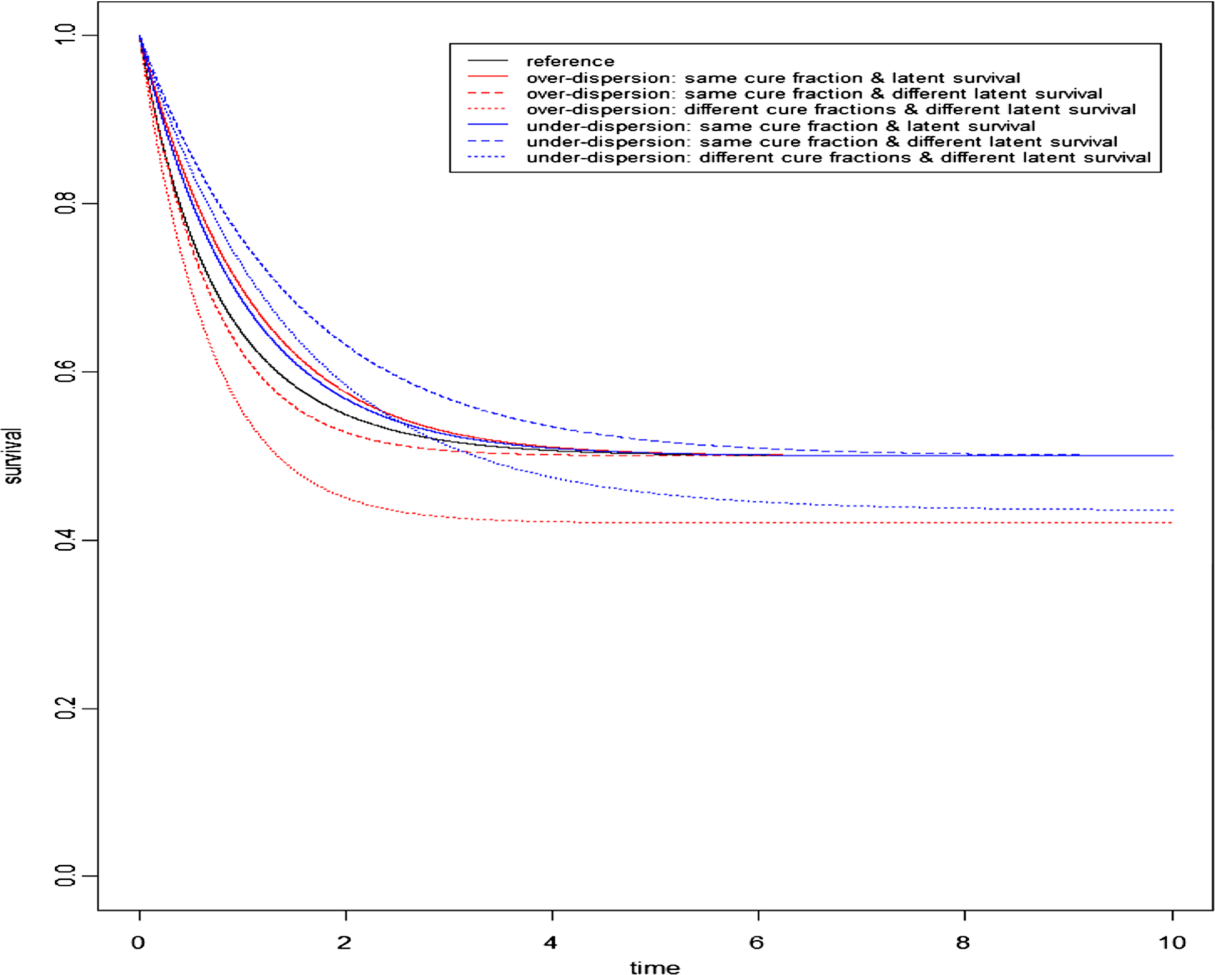
**Theoretical survival curves for seven situations.** The reference curve is in black. Survival curves for over-dispersed cases (resp. under-dispersed) are in red (resp. in blue).

To illustrate these scenarios, we plotted (Figure 1) the theoretical marginal survival curves obtained for seven situations considering a baseline cure fraction of 50% (i.e. *S*_0_(*∞*_ + _)=0.5) . The marginal survival curve for group 0 (reference curve) is in black. The survival curves for over-dispersed cases (*θ*=0.78) with same cure fraction and latent survival, same cure fraction but different latent survival functions (latent survival shift: *e*^*α*^=1.5) and different cure fractions (cure fraction shift: *e*^*γ*^=1.2) and latent survival functions are in red. The survival curves for under-dispersed cases (*θ*=−1) with same cure fraction and latent survival, same cure fraction but different latent survival functions (latent survival shift: *e*^*α*^=1.5) and different cure fractions (cure fraction shift: *e*^*γ*^=1.2) and latent survival functions are in blue.

The estimated levels of the proposed test and the logrank test and under the null hypothesis of no survival difference between the two groups are within the binomial range [0.031;0.069] for either censored cases or uncensored cases whatever the level of the cure fraction. Tables 1a, 2a and 3a (resp. Tables 1b, 2b and 3b) show the results obtained for uncensored (resp. censored) cases with overdispersion whereas Tables 4a, 5a and 6a (resp. Tables 4b, 5b and 6b) show the results for uncensored (resp. censored) cases with underdispersion.

**Table 1:** Simulation results for overdispersed cases with 30% cure fraction

**Left panel (1a) uncensored cases**	**Right panel (1b) censored cases**				
*Over*/*p*_0_=30*%*	*e*^*γ*^=1	*e*^*γ*^=1.2	*Over*/*p*_0_=30*%*	*e*^*γ*^=1	*e*^*γ*^=1.2
* cens*=0*%*			* cens*=30*%*		
*LR* *e*^*α*^=1	0.12	0.57	*LR* *e*^*α*^=1	0.16	0.62
$S^{H_{0}}\;e^{\alpha }=1$	0.58	0.80	$S^{H_{0}}\;e^{\alpha }=1$	0.47	0.79
*LR* *e*^*α*^=1.25	0.22	0.69	*LR* *e*^*α*^=1.25	0.29	0.77
$S^{H_{0}}\;e^{\alpha }=1.25$	0.87	0.97	$S^{H_{0}}\;e^{\alpha }=1.25$	0.79	0.95
*LR* *e*^*α*^=1.50	0.27	0.76	*LR* *e*^*α*^=1.50	0.42	0.83
$S^{H_{0}}\;e^{\alpha }=1.50$	0.96	0.98	$S^{H_{0}}\;e^{\alpha }=1.50$	0.90	0.97

**Table 2:** Simulation results for overdispersed cases with 50% cure fraction

**Left panel (2a) uncensored cases**	**Right panel (2b) censored cases**				
*Over*/*p*_0_=50*%*	*e*^*γ*^=1	*e*^*γ*^=1.2	*Over*/*p*_0_=50*%*	*e*^*γ*^=1	*e*^*γ*^=1.2
* cens*=0*%*			* cens*=30*%*		
*LR* *e*^*α*^=1	0.07	0.27	*LR* *e*^*α*^=1	0.15	0.38
$S^{H_{0}}\;e^{\alpha }=1$	0.38	0.57	$S^{H_{0}}\;e^{\alpha }=1$	0.28	0.48
*LR* *e*^*α*^=1.25	0.09	0.35	*LR* *e*^*α*^=1.25	0.21	0.55
$S^{H_{0}}\;e^{\alpha }=1.25$	0.69	0.83	$S^{H_{0}}\;e^{\alpha }=1.25$	0.48	0.69
*LR* *e*^*α*^=1.50	0.08	0.41	*LR* *e*^*α*^=1.50	0.29	0.66
$S^{H_{0}}\;e^{\alpha }=1.50$	0.84	0.94	$S^{H_{0}}\;e^{\alpha }=1.50$	0.63	0.83

**Table 3:** Simulation results for overdispersed cases with 70% cure fraction

**Left panel (3a) uncensored cases**	**Right panel (3b) censored cases**				
*Over*/*p*_0_=70*%*	*e*^*γ*^=1	*e*^*γ*^=1.2	*Over*/*p*_0_=70*%*	*e*^*γ*^=1	*e*^*γ*^=1.2
* cens*=0*%*			* cens*=30*%*		
*LR* *e*^*α*^=1	0.07	0.15	*LR* *e*^*α*^=1	0.12	0.20
$S^{H_{0}}\;e^{\alpha }=1$	0.29	0.33	$S^{H_{0}}\;e^{\alpha }=1$	0.14	0.27
*LR* *e*^*α*^=1.25	0.07	0.19	*LR* *e*^*α*^=1.25	0.14	0.31
$S^{H_{0}}\;e^{\alpha }=1.25$	0.40	0.54	$S^{H_{0}}\;e^{\alpha }=1.25$	0.16	0.39
*LR* *e*^*α*^=1.50	0.06	0.21	*LR* *e*^*α*^=1.50	0.21	0.42
$S^{H_{0}}\;e^{\alpha }=1.50$	0.64	0.70	$S^{H_{0}}\;e^{\alpha }=1.50$	0.22	0.48

**Table 4:** Simulation results for underdispersed cases with 30% cure fraction

**Left panel (4a) uncensored cases**	**Right panel (4b) censored cases**				
*Under*/*p*_0_=30*%*	*e*^*γ*^=1	*e*^*γ*^=1.2	*Under*/*p*_0_=30*%*	*e*^*γ*^=1	*e*^*γ*^=1.2
* cens*=0*%*			* cens*=30*%*		
*LR* *e*^*α*^=1	0.08	0.06	*LR* *e*^*α*^=1	0.15	0.05
$S^{H_{0}}\;e^{\alpha }=1$	0.34	0.45	$S^{H_{0}}\;e^{\alpha }=1$	0.27	0.31
*LR* *e*^*α*^=1.25	0.17	0.07	*LR* *e*^*α*^=1.25	0.31	0.14
$S^{H_{0}}\;e^{\alpha }=1.25$	0.73	0.81	$S^{H_{0}}\;e^{\alpha }=1.25$	0.53	0.58
*LR* *e*^*α*^=1.50	0.29	0.09	*LR* *e*^*α*^=1.50	0.48	0.23
$S^{H_{0}}\;e^{\alpha }=1.50$	0.94	0.95	$S^{H_{0}}\;e^{\alpha }=1.50$	0.76	0.75

**Table 5:** Simulation results for underdispersed cases with 50% cure fraction

**Left panel (5a) uncensored cases**	**Right panel (5b) censored cases**				
*Under*/*p*_0_=50*%*	*e*^*γ*^=1	*e*^*γ*^=1.2	*Under*/*p*_0_=50*%*	*e*^*γ*^=1	*e*^*γ*^=1.2
* cens*=0*%*			* cens*=30*%*		
*LR* *e*^*α*^=1	0.05	0.07	*LR* *e*^*α*^=1	0.07	0.07
$S^{H_{0}}\;e^{\alpha }=1$	0.13	0.17	$S^{H_{0}}\;e^{\alpha }=1$	0.08	0.10
*LR* *e*^*α*^=1.25	0.06	0.08	*LR* *e*^*α*^=1.25	0.10	0.05
$S^{H_{0}}\;e^{\alpha }=1.25$	0.34	0.39	$S^{H_{0}}\;e^{\alpha }=1.25$	0.18	0.15
*LR* *e*^*α*^=1.50	0.09	0.05	*LR* *e*^*α*^=1.50	0.11	0.10
$S^{H_{0}}\;e^{\alpha }=1.50$	0.60	0.68	$S^{H_{0}}\;e^{\alpha }=1.50$	0.31	0.28

**Table 6:** Simulation results for underdispersed cases with 70% cure fraction

**Left panel (6a) uncensored cases**	**Right panel (6b) censored cases**				
*Under*/*p*_0_=70*%*	*e*^*γ*^=1	*e*^*γ*^=1.2	*Under*/*p*_0_=70*%*	*e*^*γ*^=1	*e*^*γ*^=1.2
* cens*=0*%*			* cens*=30*%*		
*LR* *e*^*α*^=1	0.06	0.08	*LR* *e*^*α*^=1	0.05	0.08
$S^{H_{0}}\;e^{\alpha }=1$	0.05	0.09	$S^{H_{0}}\;e^{\alpha }=1$	0.07	0.07
*LR* *e*^*α*^=1.25	0.05	0.06	*LR* *e*^*α*^=1.25	0.06	0.05
$S^{H_{0}}\;e^{\alpha }=1.25$	0.10	0.15	$S^{H_{0}}\;e^{\alpha }=1.25$	0.08	0.07
*LR* *e*^*α*^=1.50	0.05	0.06	*LR* *e*^*α*^=1.50	0.09	0.05
$S^{H_{0}}\;e^{\alpha }=1.50$	0.21	0.31	$S^{H_{0}}\;e^{\alpha }=1.50$	0.10	0.06

For uncensored cases, the power gains of the proposed test are striking for either differences in cure fraction or latent survival distribution. Gains of power of the proposed test are in decreasing order of the cure fraction. In any case, the power of the proposed test is higher of those of the logrank test. For the censored case, theses latter trends are also noticed. The main difference relative to the uncensored case is in the magnitude of the power values which are more markedly decreased. In any case, the same patterns are observed for the overdispersed and underdispersed cases.

### Lung adenocarcinoma example

In early-stage lung cancer (stage I), surgical resection can be considered as effective at eliminating the tumor burden for a non-negligeable proportion of patients whereas, for the others, it leads to a lower tumor burden and thereby prolonged survival. The majority of tumor recurrences are detected within two years after the surgical resection and the five-year survival following the diagnosis is frequently considered as a cure, the main threats being other smoking-related diseases such as cardiopulmonary disorders.

The dataset considered in this study is based on a homogeneous series of 134 patients with stage IB lung adenocarcinomas who underwent surgical resection. All specimens underwent pathological review. Here, we investigated the prognostic impact of three different types of markers : genetic (Kras exon 2 mutation), genomic (recurrent copy-number losses on genomc areas 19p13.3 and 19p13.11) and histopathologic (combined marker: necrosis and differentiation).

We recalled that Kras gene belongs to a gene family of small G proteins, anchored on the cytoplasmic side of cell membrane, that play a central role in cell signalling related to cell proliferation, cell survival and cell motility (for a review see [15]). Activating mutations of Kras, which lock the protein in the active conformation, have been described in numerous epithelial tumors including lung adenocarcinomas. In a previous study ([16]), we have identified two recurrent driver copy-number losses located on the short arm of chromosome 19 (19p13.3, 19p13.11) that were exclusively deleted in lung adenocarcinomas from western european population (as compared with east-asian populations). Their prognostic impact have not been previously investigated. The prognostic impact of histopathological features of lung adenocarcinoma such as necrosis and tumor differentiation has been widely debated in the literature but recent studies pointed out that patients having tumor with necrosis or solid pattern (poorly differenciated) have an unfavorable prognosis and may be candidate for adjuvant therapy ([17]). Here, we investigated the prognostic impact of a simple histopathological marker that combines information about necrosis and differentiation level (necrosis associated with a poor differentiation versus no necrosis or well differentiated).

All patients were genotyped for Kras mutations. Primers (Kras exon 2) were used to amplify the relevant regions and DNA sequencing was performed on an ABI3730xl Sanger sequencer. All mutations were confirmed by bidirectional sequencing. In this study, the percentage of Kras mutation was 18% (24 cases), 37.6% and 34% displayed copy loss on 19p13.3 and 19p13.11, respectively, and 23% of the tumor samples showed necrosis associated with a poor differentiation. The time-to-event (death) was calculated from the date of treatment to the time of death or last follow-up. Overall survival rates were derived from Kaplan-Meier estimates and given with their 95% confidence intervals. The median of follow-up was of four years and we observed thirty sevent events. For the entire population, overall survival at two years and five years was of 87.2% [81.5-93.3] and 65.4% [56.3-75.9].

**Figure 2:**
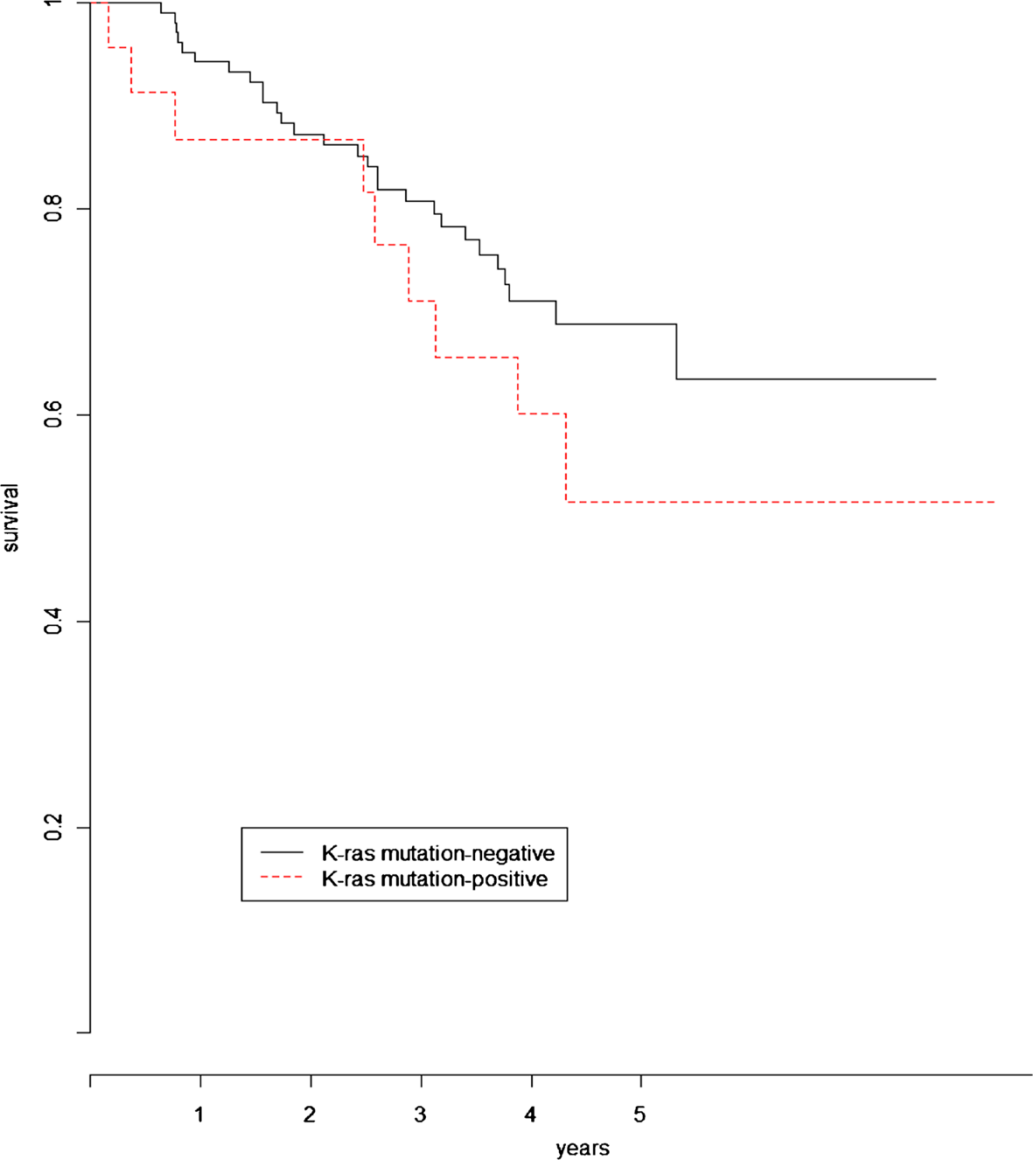
Kaplan-Meier curves of the overall survival based on Kras mutation status.

When testing for differences in overall survival for Kras mutation, the logrank test (*LR*=1.2,*p*=0.26) was not significant in contrast with the proposed test $\left(S_{H_{0}}=9.3,p=0.025\right)$. Figure 2 display the Kaplan-Meier estimates of the survival according to Kras mutation status.

**Figure 3:**
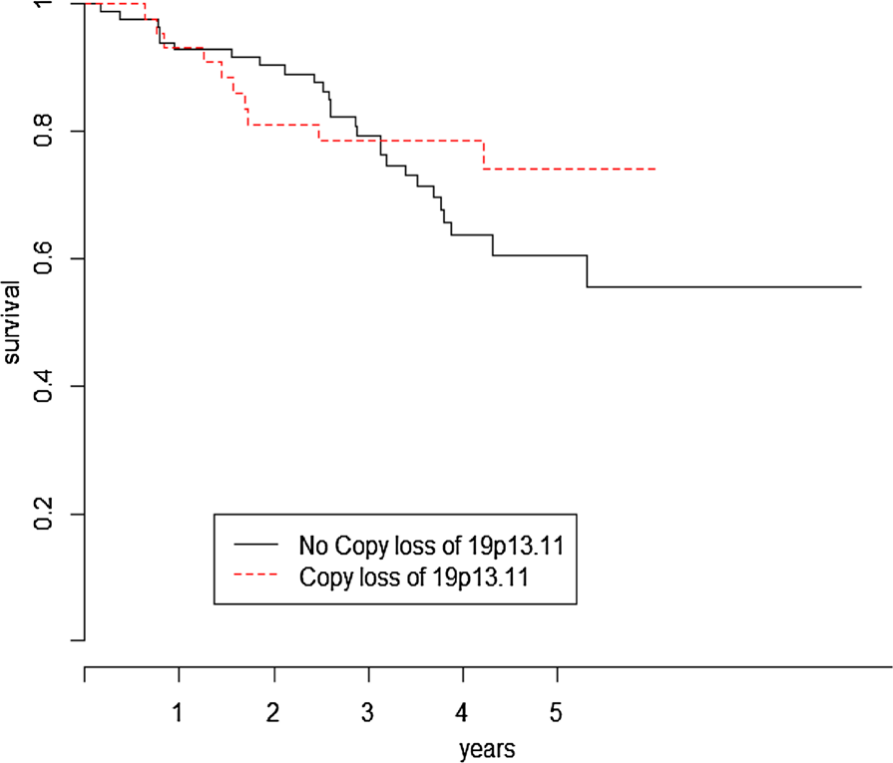
Kaplan-Meier curves of the overall survival based on copy-number loss of 19p13.11 status.

When testing for differences in overall survival for copy-number loss on genomic areas 19p13.3 and 19p13.11, the logrank test was not significant for the two areas (*LR*_19*p*13.3_=0.5,*p*=0.48;*LR*_19*p*13.11_=1,*p*=0.33) whereas the proposed test showed no difference for 19p13.3 $\left(S_{H_{0}}=4.3,p=0.23\right)$ but a significant difference for 19p13.11 $\left(S_{H_{0}}=8.2,p=0.041\right)$. Figure 3 display the Kaplan-Meier estimates of the survival according to copy-number loss on 19p13.11.

**Figure 4:**
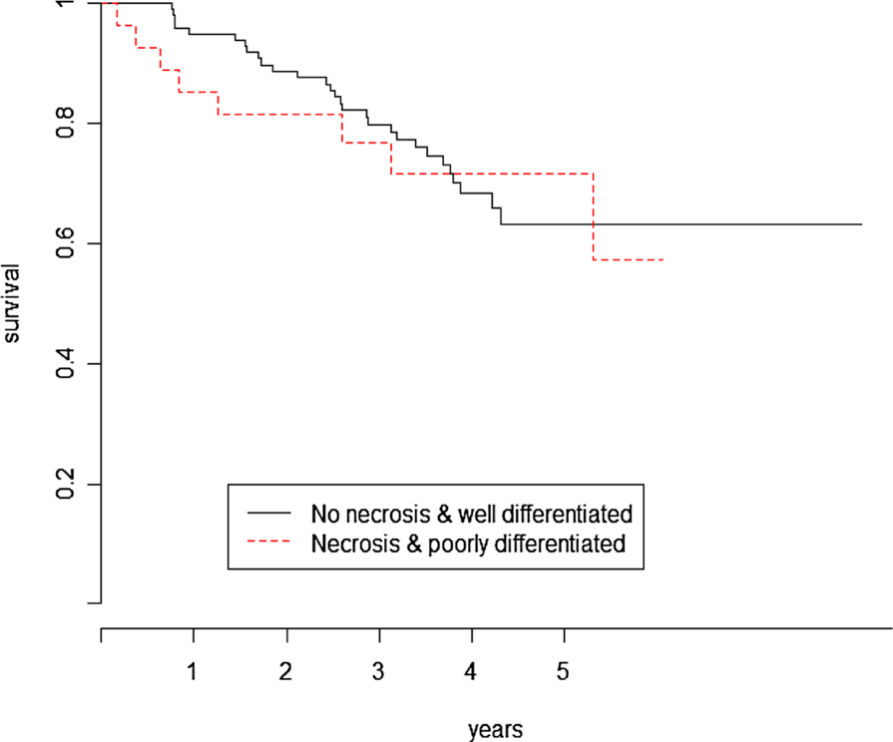
Kaplan-Meier curves of the overall survival based on the combined histopathological marker.

When testing for differences in overall survival for the combined histopathological marker, the logrank test (*LR*=0.1,*p*=0.81) was not significant in contrast with the proposed test $\left(S_{H_{0}}=7.9,p=0.048\right)$. Figure 4 display the Kaplan-Meier estimates of the survival according to the combined histopathological marker status.

All the figures show a clear time-varying effect between the two curves as time goes on. From a biological perspective, the marginal survival distribution observed for the Kras positive (activating) mutation, deletion of genomic area 19p13.11 and necrosis/poor differentiation status can be interpreted as reflecting molecular changes affecting either the tumor burden or the dynamic growth.

## Discussion

With significant progress in defining homogeneous histological and clinical group of early-stage cancer patients who sustained a same potential curative therapy, the challenge is now to find novel molecular markers having capability to separate patients according to their time-to-event outcome. This problem can be handled by considering cure rate models that are specified using either a two-component mixture model or bounded cumulative hazard approach.

In this work, a score test is proposed for testing the null hypothesis of no survival difference in early-stage of cancer. From a biological point of view, this score test allows to detect changes in the cure fraction, the distribution of surviving clonogens and the tumor progression. It is derived from a flexible model that describes the impact of discrete markers on the survival time distribution with or without a same cure fraction and stems from biological as well as pragmatic statistical considerations. A nice feature of the proposed score-type statistic is that it can be easily implemented since it does not require to estimate the parameters of the cure model under the alternative hypothesis. It should be noted that the proposed procedure can be extended for comparing more than two groups with Poisson cure rate model as the benchmark model for the reference group. The new alternative hypothesis will be such as there is at least one of the groups that differs from the reference one at some time for either the distribution of the number of clonogenes or the latent (clonogenic) survival functions.

Simulation results show that striking gains in power can be achieved by our proposed test as compared to the classical Log-rank test. As the cure rate fraction increases, the power of the test decreases, but remains higher than that of the logrank test. This latter result is not surprising, since increasing the cure fraction reduces the number of potential events. In the presence of censoring, the power of the proposed test decreases, but remains higher than that of the logrank test. It is worth recalling that the validity of the proposed score test requires asymptotic efficiency of cumulative hazard rate estimators which implies that the susceptible patients should experience the event within the maximum length of follow-up.

In our homogeneous series of early-stage lung adenocarcinoma presented in this article, the proposed statistic is particularly appealing since the majority of the patients are amenable to cure. If some lung cancer studies have reported a deleterious prognostic effect of Kras mutation, there is still some debate. In this study, we show a significant relationship between overall survival and Kras mutation status that would have been overlooked by only considering results from the classical logrank test. From a biological point of view, one could hypothesize that downstream effectors of Kras mutation have complex biological activities affecting either the tumor burden or the dynamic growth. Moreover, these results also argue in favor of considering combined histopathological marker in prognostic studies and give some interesting insights regarding recurrent driver copy-number loss on genomic area 19p13.11 that may require future exploration. In further works, it could be of interest to estimate the parameters that are associated to survival differences. For such purpose, the estimation procedure introduced by Tsodikov [18] could be envisaged.

## Conclusion

In summary, detecting molecular markers associated with complex survival patterns in early-stage cancer is of potential interest for research in enlighting their contribution to the natural history of tumor disease. We believe that our proposed score test statistic is a powerful tool for detecting molecular markers associated with complex survival patterns. Moreover, it should be noted that this test statistic can be applied in any other medical fields for which there is the possibility that some patients will not experience the event of interest.

## Competing interests

The authors declare that they have no competing interests.

## Author’s contributions

PB and TM developed the mathematical model and wrote the paper. Both authors read and approved the final manuscript.
